# Use of Single-Layer g-C_3_N_4_/Ag Hybrids for Surface-Enhanced Raman Scattering (SERS)

**DOI:** 10.1038/srep34599

**Published:** 2016-09-30

**Authors:** Jizhou Jiang, Jing Zou, Andrew Thye Shen Wee, Wenjing Zhang

**Affiliations:** 1 SZU-NUS Collaborative Innovation Center for Optoelectronic Science & Technology, Key Laboratory of Optoelectronic Devices and Systems of Ministry of Education and Guangdong Province, College of Optoelectronic Engineering, Shenzhen University, Shenzhen 518060, China; 2 Department of Physics, National University of Singapore, 2 Science Drive 3, 117542, Singapore; 3 School of Chemistry and Environmental Engineering, Key Laboratory for Green Chemical Process of Ministry of Education, Wuhan Institute of Technology, Wuhan 430073, P.R. China

## Abstract

Surface-enhanced Raman scattering (SERS) substrates with high activity and stability are desirable for SERS sensing. Here, we report a new single atomic layer graphitic-C_3_N_4_ (S-g-C_3_N_4_) and Ag nanoparticles (NPs) hybrid as high-performance SERS substrates. The SERS mechanism of the highly stable S-g-C_3_N_4_/Ag substrates was systematically investigated by a combination of experiments and theoretical calculations. From the results of XPS and Raman spectroscopies, it was found that there was a strong interaction between S-g-C_3_N_4_ and Ag NPs, which facilitates the uniform distribution of Ag NPs over the edges and surfaces of S-g-C_3_N_4_ nanosheets, and induces a charge transfer from S-g-C_3_N_4_ to the oxidizing agent through the silver surface, ultimately protecting Ag NPs from oxidation. Based on the theoretical calculations, we found that the net surface charge of the Ag atoms on the S-g-C_3_N_4_/Ag substrates was positive and the Ag NPs presented high dispersibility, suggesting that the Ag atoms on the S-g-C_3_N_4_/Ag substrates were not likely to be oxidized, thereby ensuring the high stability of the S-g-C_3_N_4_/Ag substrate. An understanding of the stability mechanism in this system can be helpful for developing other effective SERS substrates with long-term stability.

Surface-enhanced Raman scattering (SERS) is a powerful spectroscopy technique for molecular detection and characterization that relies on the enhanced Raman scattering of molecules that are adsorbed on, or near, SERS-active surfaces, such as nanostructured gold or silver^1^,^2^. Owing to its ability to achieve highly sensitive detection at the molecular level and provide non-destructive unique vibrational fingerprint information for analytes^3^,^4^, the application of SERS has greatly increased in diversity, branching out into food safety and security^5^,^6^, explosives and narcotics detection^7^,^8^,^9^, bioanalysis^10^,^11^, medicine applications^12^, clinical diagnostic and therapeutic aspects^13^,^14^, and so on. It is now well established that both a long-range electromagnetic enhancement (EM) effect and a short-range chemical enhancement (CM) effect are simultaneously operative in SERS^15^. When properly matched to the nanoscale geometries and materials that define the SERS substrates, the laser light can excite localized surface plasmons on the metal, such as gold, silver and copper, either with roughened surfaces or in the form of nanoparticles. This creates a significant localized electromagnetic field. By placing molecules in the strong electromagnetic fields, the inherently weak Raman scattering cross sections can be dramatically enhanced (by a factor of about 10^6^–10^8^). In the CM mechanism, molecules adsorbed at certain surface sites are believed to induce a charge-transfer or form σ or π bonds between the chemisorbed molecules with the metal surfaces, leading to a large change of polarizability in the chemisorbed molecules resulting in a Raman enhancement of about 10^1^–10^3^^3^,^12^.

Since the SERS substrate material and geometry play such key roles in the surface enhancement phenomenon, a great deal of research effort has been devoted to the development and characterization of superior SERS substrates. Arguably the most popular substrate comprise of colloidal nanoparticles (NPs) since they are easily prepared by the reduction of Au, Ag and Cu salt solutions and their sizes and geometries can be controlled by optimising experimental conditions^16^. However, Ag and Cu colloidal NPs are highly susceptible to oxidation under normal storage conditions. Their chemical instability cause enormous changes in the morphologies of Ag or Cu nanostructures, particularly at edges and corners with high surface free energies, and thus the deterioration of SERS activity^17^. Recently, several groups have attempted to address this problem. Yang and co-workers^18^ reported a strategy for depositing uniform shells of Au on the surfaces of Ag nanocubes to generate Ag@Au core-shell nanocubes with enhanced chemical stability and SERS activity, and the SERS activity of 1,4-benzenedithiol on the Ag@Au nanocubes remained constant over a period up to 7 days. Liu and co-workers^19^,^20^ prepared SERS-active Ag/Al_2_O_3_ films substrates based on electrochemical methods to improve the thermal stability and anti-aging of SERS-active Ag films. They found that about 60% of the Raman intensity of probe molecules adsorbed on the Ag/Al_2_O_3_ films persisted after aging for 60 days. Wolosiuk *et al*.^21^ demonstrated a simple substrate platform based on mesoporous oxide (TiO_2_, SiO_2_, and ZrO_2_) thin films containing Ag NPs for SERS chemical analysis, and the SERS activity variation of the nanocomposite substrates was within 10% of the original signal after storage of up to 42 months. Although these techniques can increase the stability of SERS substrates, the core-shell structure usually involve complex preparation processes and the thickness of metal shell is difficult to control. In addition, alumina, silica, and other materials decreased their sensitivity due to high optical absorption in the visible region. Thus there is still a need for a simple and effective route of preparing SERS substrates with high activity and stability.

Two-dimensional crystals are a new class of stable, highly processable materials that feature distinctive properties compared to their three-dimensional counterparts. By the virtue of its remarkable physical-chemical properties and molecular structure, graphene opens up a unique platform for SERS studies. It can be as a Raman probe, or as a substrate, or as an additive in SERS^22^. Similarly, as a typical layered two-dimensional chalcogenide material, MoS_2_ is considered as a promising supporting material to stabilize metal NPs. Wang’s group had prepared the Au@MoS_2_ nanocomposite by *in situ* growing Au NPs on MoS_2_ nanosheet’s surface and proved that the Au@MoS_2_ substrates exhibited attractive SERS performance^23^. An analogue of graphite, graphitic carbon nitride (g-C_3_N_4_) can be regarded as an N-substituted graphite framework consisting of π-conjugated graphitic planes formed via sp^2^ hybridization of carbon and nitrogen atoms. Recently, due to its unique electronic, optical and catalytic properties, g-C_3_N_4_ has been applied in some new promising areas, such as visible-light-induced photocatalytic hydrogen generation^24^, self-catalytic membrane photo-reactor^25^, bioimaging^26^,^27^ and CO_2_ capture^28^. We have recently explored the electronic structures of single-, bi- and few-layer g-C_3_N_4_ nanosheets by a combination of the first-principles calculations and normal Raman techniques, and unveiled a clear correlation between the spectral properties and the number of layers^29^. Based on our experiences, the combination of single atomic layer g-C_3_N_4_ (S-g-C_3_N_4_) and Ag NPs hybrids could make high SERS performance. Firstly, the Ag NPs possess a strong SERS activity and S-g-C_3_N_4_ can concentrate the target molecules, due to the π-π interaction between aromatic molecules and S-g-C_3_N_4_, which both could make the S-g-C_3_N_4_/Ag hybrid substrates display a strong SERS activity. Secondly, a strong interaction between Ag NPs and S-g-C_3_N_4_ through Ag-N bonding might bring out the Ag NPs immobilized on the surface and edges of S-g-C_3_N_4_ nanosheets becoming less susceptible to oxidation, ultimately making the S-g-C_3_N_4_/Ag hybrid substrate with a high stability.

Hence, the primary goal of this work is to prepare S-g-C_3_N_4_ and Ag NPs hybrid as a high-performance SERS substrate. The heterostructure of the S-g-C_3_N_4_/Ag hybrid was confirmed by TEM image, XRD and EDS analyses, XPS and Raman spectroscopies. Compared with Ag NPs colloid substrates, the S-g-C_3_N_4_/Ag substrates exhibited much improved long-term stability at room temperature. Furthermore, we have elucidated the impact of interface interaction on the S-g-C_3_N_4_/Ag substrates, and the origin of the stability of the S-g-C_3_N_4_/Ag substrates by a combination of experimental techniques and theoretical calculations. This work can provide a useful resource for further optimization of SERS substrates with high stability.

## Results and Discussion

### Characterization of S-g-C_3_N_4_/Ag Hybrids

**Figure 1:**
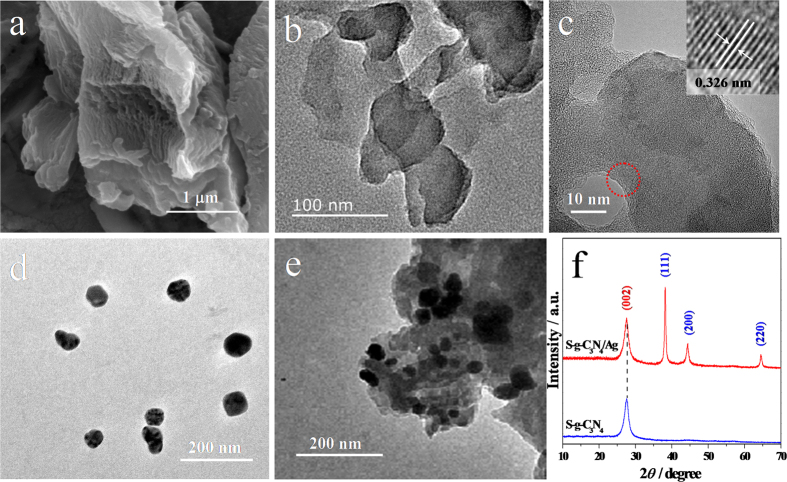
SEM image of bulk g-C_3_N_4_ (**a**), TEM images of S-g-C_3_N_4_ (**b**,**c**), Ag NPs colloid (**d**), S-g-C_3_N_4_/Ag hybrid (**e**), and XRD patterns of S-g-C_3_N_4_ and S-g-C_3_N_4_/Ag (**f**). The inset of (**c**) was HRTEM image of the as-prepared S-g-C_3_N_4_ in the delimited area of (**c**).

The bulk g-C_3_N_4_ was prepared by polymerization of melamine under an air atmosphere^1^. The SEM image (Fig. 1a) shows that the product obtained displays micrometer-size sheet-like structures. To analyze the chemical composition, XPS analysis (Figure S1 in the Supporting Information) showed that the synthesized bulk g-C_3_N_4_ was mainly composed of C and N elements, and the molar ratio of N/C was about 1.44, close to the stoichiometric ratio of C_3_N_4_. These results indicate that the micrometer-size sheet-like g-C_3_N_4_ had been successfully prepared. Single atomic layer g-C_3_N_4_ (S-g-C_3_N_4_) nanosheets were obtained by a simple chemical exfoliation of the bulk g-C_3_N_4_^29^. The typical TEM image of S-g-C_3_N_4_ nanosheets is shown in Fig. 1b, indicating the ultrathin thickness of the S-g-C_3_N_4_ nanosheets. The darker part in the TEM image can be attributed to the overlap of several S-g-C_3_N_4_ nanosheets. Figure 1c shows a high resolution TEM (HR-TEM) image of S-g-C_3_N_4_ nanosheets. A lattice plane separation of 0.326 nm that corresponds to the inter-layer distance, indexed for the (002) crystallographic plane of g-C_3_N_4_^30^, can be seen in the inset of Fig. 1c.

Meanwhile, the TEM image of Ag NPs (Fig. 1d) clearly displays the as-prepared Ag NPs colloid were monodisperse with an average diameter of about 40 nm, being in agreement with the result obtained from the size distribution of Ag NPs colloid (41.78 nm, Figure S2 in the Supporting Information). Figure 1e shows a TEM image of the S-g-C_3_N_4_/Ag NPs hybrid. It was seen that Ag NPs were uniformly immobilized on the edges and surface of S-g-C_3_N_4_ nanosheets, which implied the S-g-C_3_N_4_/Ag NPs hybrids with good uniformity were successfully synthesized. In addition, the chemical composition of obtained S-g-C_3_N_4_/Ag NPs hybrid was determined using EDS technique, which is shown in Figure S3 in the Supporting Information. The obvious signals for C, N and Ag elements marked in Figure S3. This EDS analysis further confirmed the successful synthesis of S-g-C_3_N_4_/Ag hybrid.

Moreover, the S-g-C_3_N_4_ and S-g-C_3_N_4_/Ag NPs hybrids were also characterized by XRD, as shown in Fig. 1f. In the XRD pattern of S-g-C_3_N_4_, there was only a (002) peak at 2θ = 27.3°, which reflected the characteristic interlayer stacking structure, consistent with the HR-TEM result. The (100) diffraction at 13.1° was related to the interplanar structural packing. And almost invisible peak was observed around 13.1°, indicating that the S-g-C_3_N_4_ was successfully synthesized^30^. In the XRD pattern of the S-g-C_3_N_4_/Ag NPs hybrid, three additional diffraction peaks were observed. They are assigned to the (111), (200) and (220) peaks of cubic Ag crystal (JCPDS Card No. 04–0783), suggesting the successfully preparation of the S-g-C_3_N_4_/Ag hybrid.

### High SERS Activity of S-g-C_3_N_4_/Ag Substrates

To investigate the SERS activity of the S-g-C_3_N_4_/Ag substrates, crystal violet (C_25_H_30_N_3_Cl·_9_H_2_O, CV) molecule with a propeller-like shape and D_3_ symmetry was used as the probe molecule in subsequent experiments. It was found that there was a strong absorption around 589 nm in the UV-vis absorption spectrum of CV solution (Figure S4 in the Supporting Information). This allows much easier occurrence of resonance enhancement Raman of CV molecules if an incident laser with 532 nm was used. Moreover, a strong absorption peak at 415 nm was observed in the UV-vis absorption spectrum of the S-g-C_3_N_4_/Ag suspension (Figure S5 in the Supporting Information). It is known that when the frequency of incident light is resonant with a plasmon from the metal nanoparticles, it leads to redistribution of the local field and a great enhancement of the electromagnetic field at a specific position around the nanoparticles (called a “hot spot”). Hence, among the commonly used incident lasers with 532 nm, 633 nm and 785 nm, the frequency of incident light of the laser with 532 nm is easiest resonant with the plasmon from the S-g-C_3_N_4_/Ag substrates. Therefore, we choose CV as the probe molecule and an excitation laser of 532 nm as the incident light source to investigate the SERS activities of substrates in our experiments.

**Figure 2:**
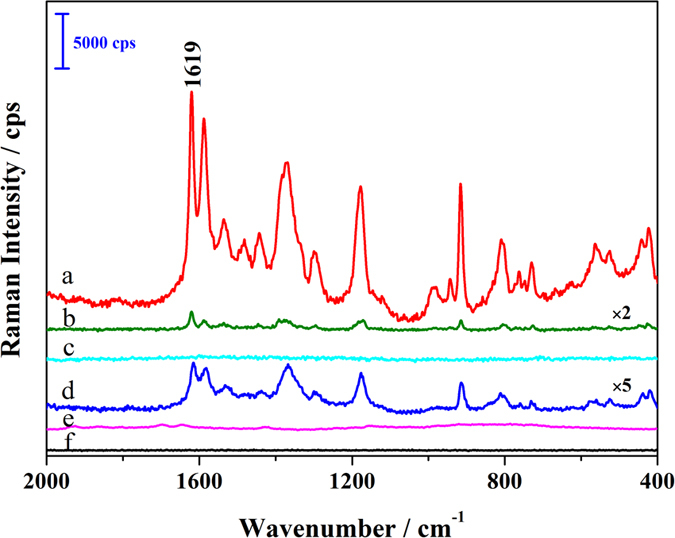
SERS spectra of CV (2.5 × 10^−6^ mol L^−1^) on S-g-C_3_N_4_/Ag (**a**), Ag NPs colloid (**b**) and S-g-C_3_N_4_ (**c**). The normal Raman spectra of CV powder (**d**), S-g-C_3_N_4_ (**e**) and CV solution (2.5 × 10^−6^ mol L^−1^) (**f**) were given for comparison. The Raman measured conditions are: an excitation laser of 532 nm with, a power of 1 mW, the sample exposure times of 1, and the collect exposure time of 0.2 s.

Figure 2 shows the SERS spectra of CV solution (2.5 × 10^−6^ mol L^−1^) on S-g-C_3_N_4_/Ag (Fig. 2a), Ag NPs colloid (Fig. 2b) and S-g-C_3_N_4_ (Fig. 2c) substrates, respectively. The normal Raman spectrum of CV powder (Fig. 2d) was used as the reference for the peak position in SERS measurement. The normal Raman spectra of S-g-C_3_N_4_ (Fig. 2e) and CV solution (2.5 × 10^−6^ mol L^−1^) (Fig. 2f) were given for comparison.

No peaks are observed in the normal Raman spectrum of CV solution (Fig. 2f), but the normal Raman spectrum of CV powder (Fig. 2d) shows plentiful peaks. Negligible SERS responses of CV were observed on the S-g-C_3_N_4_ substrates (Fig. 2c), and very weak responses with limited CV characteristic peaks were produced on the Ag NPs colloid substrates (Fig. 2b). However, all the characteristic peaks of CV molecules were significantly enhanced in intensity in the case of the SERS spectrum of the S-g-C_3_N_4_/Ag substrates (Fig. 2a), demonstrating that there was a significant Raman enhancement effect on the S-g-C_3_N_4_/Ag substrates surface. According to the relevant calculations of the enhancement factor^31^,^32^, the S-g-C_3_N_4_/Ag substrates exhibited an enhancement factor as high as 2.1 × 10^9^, being much higher than that provided by individual Ag NPs colloid (3.1 × 10^6^) as a control. These results suggested that the S-g-C_3_N_4_/Ag substrates can work effectively for SERS sensoring.

### High Stability of S-g-C_3_N_4_/Ag Substrates

**Figure 3:**
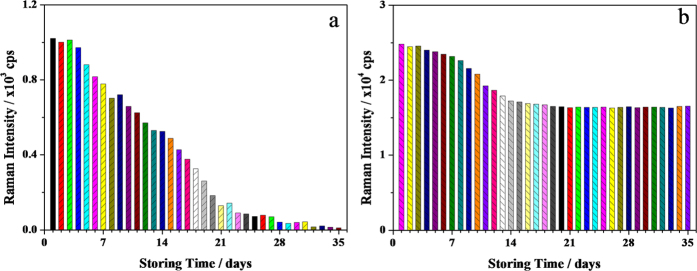
The relationships between SERS intensity and storing time of substrate materials of Ag NPs colloid (**a**) and S-g-C_3_N_4_/Ag (**b**), respectively. The peak intensity at 1619 cm^−1^ of CV was used as a reference. The SERS measured conditions are: an excitation laser of 532 nm with, a power of 1 mW, the sample exposure times of 1, and the collect exposure time of 0.2 s.

Generally speaking, a good SERS substrate needs relatively high stability during use and storage. The aggregation or oxidation of Ag NPs could cause a significant decrease of the SERS activity of the substrates^33^. The stability of S-g-C_3_N_4_/Ag substrates was investigated by comparing the SERS intensity of Ag NPs colloid and S-g-C_3_N_4_/Ag substrates under different storage durations at room temperature, as shown in Fig. 3. Here, the peak intensity of CV at 1619 cm^−1^ in the SERS spectra was used as the reference. It was found that for the Ag NPs colloid, the peak intensity of CV at 1619 cm^−1^ was dramatically decreased after prolonged storage time (Fig. 3a), becoming negligible after four weeks, suggesting that Ag NPs colloid substrates were SERS inactive after four weeks storage. In contrast, the observed SERS peak intensity on the S-g-C_3_N_4_/Ag substrates decreased only slightly in the initial two weeks (Fig. 3b), and then remained almost constant for longer time periods (more than five weeks) with a relative strong peak intensity of about 17,000 cps (at 1619 cm^−1^), which was fifteen times higher than that observed on freshly prepared Ag NPs colloid substrates. This result indicated that the S-g-C_3_N_4_/Ag substrates exhibit high stability at room temperature. The dramatic decrease of SERS activity on Ag NPs colloid substrates was attributed to the oxidation and aggregation of the Ag NPs colloid during the storing process^33^. For the S-g-C_3_N_4_/Ag substrates, the essential reason for their high stability might be due to the interaction between Ag NPs and S-g-C_3_N_4_ through Ag-N bonding, resulting in Ag NPs immobilized on the surface and edges of S-g-C_3_N_4_ nanosheets and becoming less susceptible to oxidation.

### An insight into the High Stability of S-g-C_3_N_4_/Ag Substrates

#### Interaction between S-g-C_3_N_4_ and Ag in S-g-C_3_N_4_/Ag Substrates

**Figure 4:**
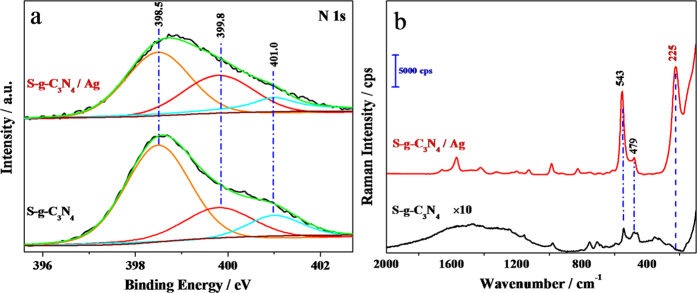
N 1s XPS spectra (**a**) and of Raman spectra (**b**) of S-g-C_3_N_4_ and S-g-C_3_N_4_/Ag, respectively. The Raman measured conditions are: an excitation laser of 780 nm with, a power of 24 mW, the sample exposure times of 30, and the collect exposure time of 0.2 s.

To investigate the essential reason for the high stability of S-g-C_3_N_4_/Ag substrates, XPS N 1s and Raman spectra of S-g-C_3_N_4_ and S-g-C_3_N_4_/Ag were analyzed. Figure 4a compares the XPS N 1s envelops of S-g-C_3_N_4_ and S-g-C_3_N_4_/Ag, which could be fitted with three peaks at 398.5, 399.8 and 401.0 eV, assigned to pyridinic N (C-N-C), pyrrolic N (N-[C]_3_) and graphitic N (C-NH), respectively^30^. In other words, these three peaks at 398.5, 399.8 and 401.0 eV correspond to sp^2^-hybridized N, sp^3^-hybridized N and amino functional groups with a hydrogen atom, respectively. It was found that the intensity ratio of N(sp^2^)/N(sp^3^) decreased from 2.52 to 1.47 after Ag NPs was deposited on S-g-C_3_N_4_. This was attributed to Ag-π interaction or η^1^ and η^2^ coordination of Ag NPs to S-g-C_3_N_4_^34^,^35^, causing some sp^2^-hybridized nitrogen atoms to form sp^3^-hybridized forms in heptazine heterocycles. The decreased N(sp^2^)/N(sp^3^) ratio in the XPS N 1 s profiles confirms the interaction between S-g-C_3_N_4_ and Ag. Moreover, we postulate that the oxygen reduction reaction (ORR) active site is created by pyridinic N involved in the triazine rings (C-N-C), and the ORR activity of the catalyst is strongly dependent on the concentration of pyridinic N^36^. It is noted that the peak area at 398.5 eV of S-g-C_3_N_4_/Ag substrates decreased compared with that of S-g-C_3_N_4_. In other words, the concentration of pyridinic N in the S-g-C_3_N_4_/Ag substrates was lower than that of S-g-C_3_N_4_, which suggests the ORR activity decreased after interaction with Ag NPs. This result implies that in comparison with S-g-C_3_N_4_, the S-g-C_3_N_4_/Ag substrates are more inert in ambient air, and there is a strong interaction between S-g-C_3_N_4_ and Ag NPs forming a stable hybrid structure.

Figure 4b shows the normal Raman spectra of S-g-C_3_N_4_ and S-g-C_3_N_4_/Ag. Several characteristic peaks were observed at 1555, 751, 705, 543, and 479 cm^−1^ for S-g-C_3_N_4_, corresponding to the vibration modes of CN heterocycles^29^. We carried out first principles calculations to identify the corresponding Raman vibrational modes of g-C_3_N_4_. According to the calculations, the peaks around 543 and 479 cm^−1^ are ascribed to in-plane symmetrical stretching and the twisting vibration of heptazine heterocycles (Figure S6 in the Supporting Information), respectively. By comparing the Raman spectra of the S-g-C_3_N_4_ and S-g-C_3_N_4_/Ag, all the mentioned characteristic peaks are also detected on S-g-C_3_N_4_/Ag. Moreover, the peaks at 543 and 479 cm^−1^ are significantly enhanced in intensity in the Raman spectrum of S-g-C_3_N_4_/Ag, which can be attributed to the SERS effect of Ag NPs. Furthermore, in the spectrum of S-g-C_3_N_4_/Ag, there is a new strong peak at 225 cm^−1^, which is attributed to the N-Ag symmetric stretching mode, in accordance with the reported results^37^,^38^. The appearance of this new peak also supports our hypothesis of an interaction between S-g-C_3_N_4_ and Ag through the N-Ag bonding. Therefore, we conclude that this strong interaction serves to uniformly immobilize Ag NPs on the edges and surface of S-g-C_3_N_4_ nanosheets to protect Ag NPs from oxidation, as well as to form a more stable structure, ensuring the stability of the S-g-C_3_N_4_/Ag substrate ambient storage conditions.

#### Charge Transfer between S-g-C_3_N_4_ and Ag

**Figure 5:**
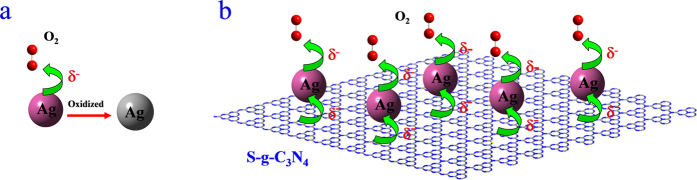
Schematic illustration of the charge transfer process among S-g-C_3_N_4_, O_2_ and Ag . The label δ^−^ denotes the negative charge of Ag surface or S-g-C_3_N_4_.

Silver has 4d^10^5s^1^ valence electron configuration and commonly coordinates through its sp hybrid orbitals. The N atom of g-C_3_N_4_ can donate its lone pair of electrons to the metal atom and its 3d-orbitals could accept the d electrons of a transition metal in homogeneous composites^37^,^38^,^39^. For Ag NP colloid substrates, the single-electron in the surface silver atom can easily be taken away by the oxidizing agent (such as O_2_) leading to oxidation, as shown in Fig. 5a. However, for Ag NPs immobilized on the S-g-C_3_N_4_ nanosheets surface, some N atoms of S-g-C_3_N_4_ provide lone pairs of electrons to occupy the vacant sp hybrid orbitals of silver, and its 3d-orbitals could accept the d electrons of Ag atoms. Because of the poor *π*-acceptor and strong *σ*-donor ability of the N atoms and the weak d-feedback property of silver, the net charge transfer is from S-g-C_3_N_4_ to the silver surface (Fig. 5b). Hence, in the real S-g-C_3_N_4_/Ag substrate system, when O_2_ or other oxidizing agent are exposed to the S-g-C_3_N_4_/Ag substrates, the negative charge from S-g-C_3_N_4_ could transfer to the oxidizing agent through the silver surface, which could protect Ag NPs from oxidation. The schematic of the proposed charge transfer process is shown in Fig. 5b. In addition, as the atomic content of N is close to 60% in g-C_3_N_4_, there are sufficient N atoms in S-g-C_3_N_4_ substrates to provide lone pairs of electrons to prevent oxidation of Ag NPs and enhance the stability of S-g-C_3_N_4_/Ag NPs hybrid substrates.

#### Net Surface Charge of Ag in S-g-C_3_N_4_/Ag Substrates

**Figure 6:**
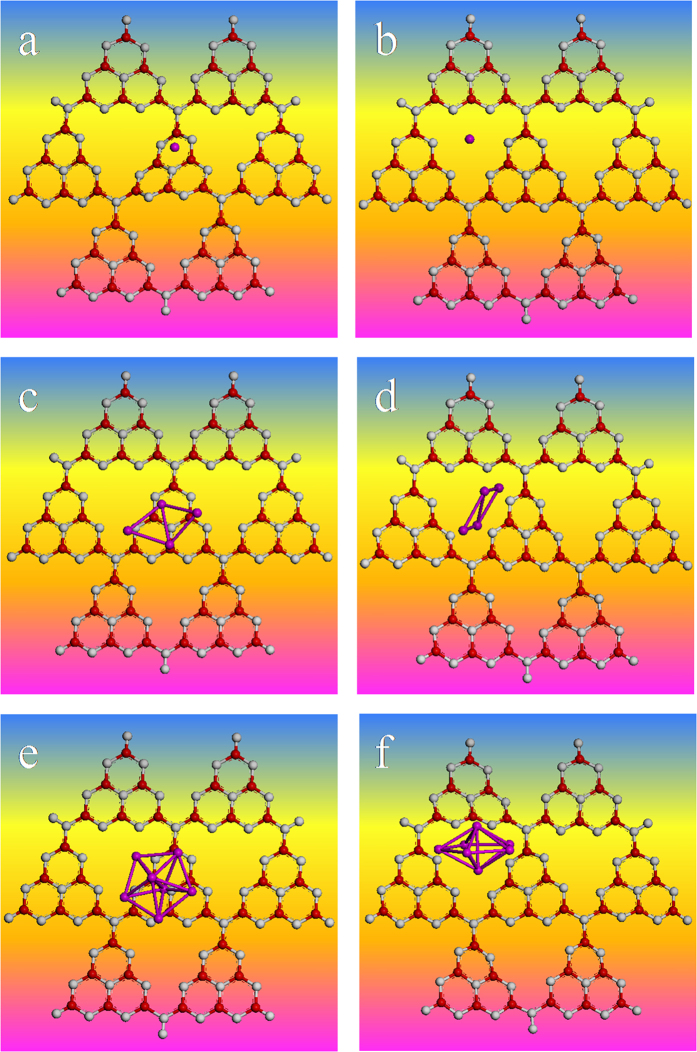
The optimized geometries of g-C_3_N_4_/Ag_n_ (n = 1) (**a**,**b**), the geometries of g-C_3_N_4_/Ag_n_ of before and after optimization: n = 4 (**c**,**d**), n = 7 (**e**,**f**), respectively. Red, gray and purple spheres represent C, N and Ag atoms, respectively.

To gain further insight into the essential reason for the high stability of S-g-C_3_N_4_/Ag substrates, the first principles calculations were performed to determine the stable optimized geometry of S-g-C_3_N_4_. Silver cluster models with different sizes of Ag_n_ (n = 1~10) were used in the CASTEP calculations to simulate Ag NPs surfaces for optimizing the structures of S-g-C_3_N_4_/Ag_n_. When the silver cluster was Ag_1_, the calculations showed that the optimized geometries of S-g-C_3_N_4_/Ag has two configurations, namely, a silver atom located in a small six-membered ring (Fig. 6a) and a silver atom stabilized in a carbon nitride pore composed of three adjacent heptazine units (Fig. 6b). As the energy of the former configuration is higher by ~1 eV, the more stable conformation for Ag_1_ is the latter (Fig. 6b). Similarly, we have also calculated the different configurations with silver clusters from Ag_2_ to Ag_10_ (the results were not all shown here). It was found that the most stable conformations did not change drastically upon varying the silver cluster size from Ag_2_ to Ag_10_, and the most stable conformations for S-g-C_3_N_4_/Ag_n_ were the silver clusters located in the nitride porous composed of three adjacent heptazine units (Fig. 6c–f).

Accordingly, the most stable conformations of silver clusters and S-g-C_3_N_4_/Ag_n_ were used in the present study to analyze the surface charge of Ag atoms. As shown in Table 1, we randomly selected several stable configurations to compare the Milliken atomic charge distributions of Ag atoms. It can be seen that the net surface charge of Ag atoms in different silver clusters are almost zero, which was consistent with the fact that pure Ag NPs are neutral. However, the net surface charges of Ag atoms in different S-g-C_3_N_4_/Ag_n_ systems are all positive, for example, in the S-g-C_3_N_4_/Ag_7_ system, the total charge of Ag atoms was up to 0.423 e. It is known that the materials with much more positive charge were more difficult to oxidize. Therefore, in comparison with pure Ag NPs, the Ag atoms with the positive net surface charge in S-g-C_3_N_4_/Ag substrates more difficult to oxidize, and this is attributed to the strong interaction between S-g-C_3_N_4_ and Ag NPs. This is probably the most direct reason for the excellent stability of S-g-C_3_N_4_/Ag substrates.

#### Dispersion of Ag in S-g-C_3_N_4_/Ag Substrates

**Figure 7:**
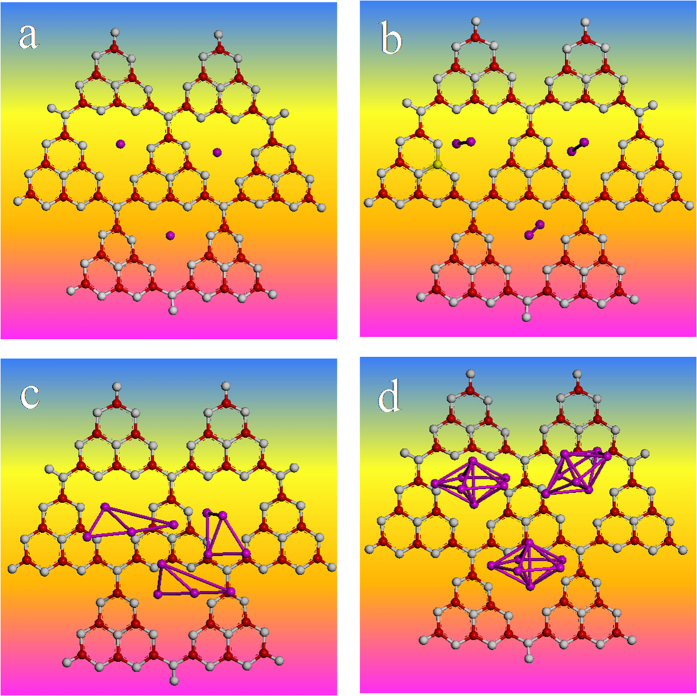
The optimized geometries of S-g-C_3_N_4_/(3-Ag_n_): (**a**) n = 1, (**b**) n = 2, (**c**) n = 4 and (**d**) n = 7, respectively.

Generally speaking, the oxidation and agglomeration processes of the metal nanoparticles occur simultaneously. Table 1 provides the charge distributions of Ag atoms in S-g-C_3_N_4_/Ag substrates and suggests that the Ag NPs immobilized on the S-g-C_3_N_4_ nanosheets surface are substantially not oxidized. If so, whether the Ag NPs in S-g-C_3_N_4_/Ag substrates were really without agglomeration? Firstly, from the TEM image of S-g-C_3_N_4_/Ag (Fig. 1e), it was easy to see that the Ag NPs were uniformly immobilized on the edges and surface of S-g-C_3_N_4_ nanosheets, rather than agglomerated. Moreover, we randomly simulated three adjacent silver clusters on the surface of S-g-C_3_N_4_ nanosheets to see what would happen. As shown in Fig. 7, in the optimized geometries of S-g-C_3_N_4_/(3-Ag_n_), the three adjacent silver clusters were almost stabilized in the corresponding nitride porous composed of three adjacent heptazine units, without any agglomeration. Therefore, TEM and first-principles calculations show that the Ag NPs in S-g-C_3_N_4_/Ag substrates exhibit excellent dispersibility, which contributes to the high stability of S-g-C_3_N_4_/Ag substrates. On the basis of the above discussions, it is found that the strong interaction and a charge transfer effect between S-g-C_3_N_4_ and Ag NPs, the relatively positive net surface charge and good dispersion of Ag NPs in S-g-C_3_N_4_/Ag substrates, all contribute to the stability of S-g-C_3_N_4_/Ag substrates.

## Conclusion

In summary, S-g-C_3_N_4_/Ag substrates with good SERS performance were successfully prepared, and they exhibit a strong Raman enhancement effect for CV probe molecules with an enhancement factor of 2.1 × 10^9^. In particular, the S-g-C_3_N_4_/Ag substrates are stable compared to Ag NPs colloid substrates during long-term storage at room temperature. We have combined experimental results (XPS, Raman and TEM) and first-principles calculations to explain the high stability of S-g-C_3_N_4_/Ag substrates. We found a strong interaction and a charge transfer effect between S-g-C_3_N_4_ and Ag NPs, which could protect Ag NPs from oxidation. The net surface charges on the Ag atoms in S-g-C_3_N_4_/Ag substrates were positive, suggesting that these Ag atoms are difficult to oxidize. In addition, we experimentally and theoretically showed that the Ag NPs in S-g-C_3_N_4_/Ag substrates display excellent dispersibility. This work demonstrates the high stability of the S-g-C_3_N_4_/Ag substrates for practical use and storage.

## Methods

### Fabrication of bulk g-C_3_N_4_

The bulk g-C_3_N_4_ was prepared by polymerization of melamine (C_3_N_3_(NH_2_)_3_) in ambient air^31^. Briefly, a ceramic crucible loaded with 4 g of melamine powder was placed into the centre of a muffle furnace. The furnace was gradually heated to 520 °C at a rate of 10 K min^−1^ and kept at this temperature for 4 h. After cooling the furnace to room temperature, a yellow-colored product was found in the downstream region of the ceramic crucible. The product obtained was characterized as bulk g-C_3_N_4_ powders and its stoichiometry determined by XPS.

### Fabrication of S-g-C_3_N_4_ nanosheets

The S-g-C_3_N_4_ nanosheets were obtained by sulfuric acid intercalation and liquid exfoliation of bulk g-C_3_N_4_ in water^29^,^30^. Typically, bulk g-C_3_N_4_ (300 mg) was dispersed in concentrated sulfuric acid (98%, 3 mL), and then rapidly agitated for 9 h. Then the mixture was slowly poured into ultrapure water under ultrasonic irradiation. The resultant product was filtered and washed repeatedly with water till neutrality. The finally product was dispersed in water.

### Fabrication of Ag NPs colloid

Ag NPs colloid were prepared by the hydroxylamine hydrochloride reduction method^32^,^37^. Typically, 40 mL of NaOH solution (7.5 mmol L^−1^) was added to 50 mL of the NH_2_OH•HCl solution (3.0 mmol L^−1^) under ultrasound irradiation. 10 mL of AgNO_3_ aqueous solution (10 mmol L^−1^) was then rapidly added to the mixture under ultrasound irradiation with a power of 140 W. After 5 min, a milky gray color colloid was obtained and stored in a refrigerator at 4 °C prior to use.

### Fabrication of S-g-C_3_N_4_/Ag hybrids

To prepare the hybrids, S-g-C_3_N_4_ nanosheet powders (0.02 g) were added in 20 mL of Ag NPs colloid under vigorous stirring at room temperature. After stirring for 1 h, the resultant dispersion was stored at room temperature prior to use. All the chemicals were of analytical grade and were used as received. Milli-Q water (18.2 MΩ·cm) provided by a Milli-Q Labo apparatus (Thermo Fischer Scientific) was used in all experiments.

### Characterization

The morphology of bulk g-C_3_N_4_ was examined with a field emission scanning electron microscope (FEI Nova NanoSEM). Transmission electron microscopic (TEM) images and the energy dispersive spectra (EDS) were recorded on a TECNAI G2 20U-Twin electron microscope, using an accelerating voltage of 200 kV. Samples for TEM analysis were prepared by drying a drop of nanocrystal dispersion in absolute ethanol on carbon-coated copper grids. The size distribution of Ag NPs colloid was characterized with a Nano-ZS90 zetasizer instrument (Malvern). UV-visible absorption spectra were recorded on a Shimadzu UV-2550 spectrophotometer (Kyoto, Japan). The crystallinity was determined on a Bruker D8 Advance TXS X-rays diffractometer (XRD) with a Cu Kα radiation (λ = 1.54 Å) source (applied voltage 40 kV, current 40 mA). Scans were recorded for 2θ values between 10° and 80°, using a step size of 0.02° and integration of 16 s per step. Raman spectra were measured on a confocal laser micro-Raman spectrometer (NT-MDT, Moscow, Russia) equipped with a diode laser of excitation of 532 nm and a diode laser of excitation of 780 nm. The laser was focused onto the sample with an Olympus ×50 long distance objective. The radius of the illumination laser spot size was ~0.55 μm. The samples were mounted on an automatic stage allowing the control of 1 μm step size. Spectra were obtained at a laser power of 1 mW (532 nm) or 24 mW (780 nm), and a 0.2 s acquisition time with 1800 lines/mm grating in the wavenumber range of 50~3500 cm^−1^. Baseline corrections were carried out to correct for the optical background signal from the substrate. Data collection, processing, and analysis were performed using the Thermo Scientific OMNIC software suite, including OMNIC™ Atlμs™. For SERS analysis, the S-g-C_3_N_4_/Ag hybrid suspension was firstly shaken for several minutes to obtain a uniform dispersion. In the SERS measurements using CV as the probe, 0.1 mL of the hybrid dispersion and 2 mL of CV solution (2.5 × 10^−6^ mol L^−1^) were mixed together, followed by shaking for 5 min. The mixture (10 μL) was finally dropped onto a clean glass slide for the measurement. Other analytes were also detected similarly. X-ray photoelectron spectroscopic (XPS) analysis was conducted on a VG Multilab 2000 spectrometer (Thermo Electron Corporation) with Al Kα radiation as the excitation source (300 W). During the measurement, the sample was supported on a copper substrate. The binding energies of recorded XPS spectra were corrected according to the C 1 s line at 284.6 eV. After subtracting the Shirley-type background, the core-level spectra were decomposed into their components with mixed Gaussian-Lorentzian (20:80) shapelines using the CasaXPS software.

### Calculations

The geometry and electronic structure of g-C_3_N_4_, silver clusters and g-C_3_N_4_/Ag hybrids, and the vibrational modes of Raman spectra for the optimized g-C_3_N_4_ structures were performed using the plane-wave ultrasoft (PWUS) pseudo-potential method as implemented in the Cambridge Sequential Total Energy Package (CASTEP) code^40^. The theoretical study was also performed using the PWUS pseudo-potential method with the generalized gradient approximation (GGA) and correlation in the Perdew-Wang 91 (PW91)^41^, and with the local density approximation (LDA) functional of Ceperley and Alder as parameterized by Perdew and Zunger (CAPZ)^42^. The valence atomic configurations are 2s^2^2p^2^ for C, 2s^2^2p^3^ for N. A cutoff energy of 400 eV and a Monkhorst-Pack k-mesh of 4 × 4 × 1 are used. The self-consistent convergence accuracy was set at 4 × 10^−6^ eV/atom. The convergence criterion for the maximal force on atoms is 0.02 eV/Å. The maximum displacement is 5 × 10^−4^ Å, and the stress is less than 0.02 GPa.

## Additional Information

**How to cite this article**: Jiang, J. *et al*. Use of Single-Layer g-C_3_N_4_/Ag Hybrids for Surface-Enhanced Raman Scattering (SERS). *Sci. Rep*. **6**, 34599; doi: 10.1038/srep34599 (2016).

## Supplementary Material

Supplementary Information

## Figures and Tables

**Table 1:** Milliken atomic charge distributions of Ag atoms in the different optimized geometries of g-C_3_N_4_/Ag_n_ and Ag_n_ systems.

Sample	Atomic Categories	Charge	Sample	Atomic Categories	Charge
S-g-C_3_N_4_/Ag	Ag (1)	0.401	Ag	Ag (1)	0.000
Total charge		**0.401**	Total charge		**0.000**
S-g-C_3_N_4_/Ag_2_	Ag (1)	0.185	Ag_2_	Ag (1)	0.000
	Ag (2)	−0.075		Ag (2)	0.000
Total charge		**0.110**	Total charge		**0.000**
S-g-C_3_N_4_/Ag_4_	Ag (1)	0.085	Ag_4_	Ag (1)	−0.147
	Ag (2)	0.051		Ag (2)	0.147
	Ag (3)	0.208		Ag (3)	0.148
	Ag (4)	−0.042		Ag (4)	−0.147
Total charge		**0.302**	Total charge		**0.001**
S-g-C_3_N_4_/Ag_7_	Ag (1)	−0.030	Ag_7_	Ag (1)	−0.026
	Ag (2)	0.172		Ag (2)	−0.025
	Ag (3)	0.025		Ag (3)	−0.026
	Ag (4)	−0.027		Ag (4)	−0.025
	Ag (5)	−0.039		Ag (5)	−0.026
	Ag (6)	0.153		Ag (6)	0.064
	Ag (7)	0.169		Ag (7)	0.064
Total charge		**0.423**	Total charge		**0.000**
S-g-C_3_N_4_/Ag_8_	Ag (1)	0.042	Ag_8_	Ag (1)	0.095
	Ag (2)	0.135		Ag (2)	−0.034
	Ag (3)	0.024		Ag (3)	−0.012
	Ag (4)	−0.066		Ag (4)	−0.035
	Ag (5)	0.021		Ag (5)	0.021
	Ag (6)	0.044		Ag (6)	−0.012
	Ag (7)	0.036		Ag (7)	0.021
	Ag (8)	0.140		Ag (8)	−0.043
Total charge		**0.376**	Total charge		**0.001**
