# Supplementary material for: Use of Single-Layer g-C3N4/Ag Hybrids for Surface-Enhanced Raman Scattering (SERS)
Source: Sci Rep. 2016 Sep 30;6:34599. doi: 10.1038/srep34599 (PMC5043347; doi:10.1038/srep34599)
Supplement: Supplementary Information [file srep34599-s1.doc]

**Supporting Information**

Use of Single-Layer g-C_3_N_4_/Ag Hybrids for Surface-Enhanced Raman Scattering (SERS)

Jizhou Jiang,^1,2^ Jing Zou,^3^ Andrew Thye Shen Wee,^2^ and Wenjing Zhang^1^,*

^1^SZU-NUS Collaborative Innovation Center for Optoelectronic Science & Technology, Key Laboratory of Optoelectronic Devices and Systems of Ministry of Education and Guangdong Province, College of Optoelectronic Engineering, Shenzhen University, Shenzhen 518060, China.

^2^Department of Physics, National University of Singapore, 2 Science Drive 3, Singapore 117542.

^3^School of Chemistry and Environmental Engineering, Key Laboratory for Green Chemical Process of Ministry of Education, Wuhan Institute of Technology, Wuhan 430073, P.R. China.

Corresponding Author

*Email: [wjzhang@szu.edu.cn](mailto:wjzhang@szu.edu.cn) (W. Zhang)

**Figure S1.** XPS spectra of synthesized bulk g-C_3_N_4_.


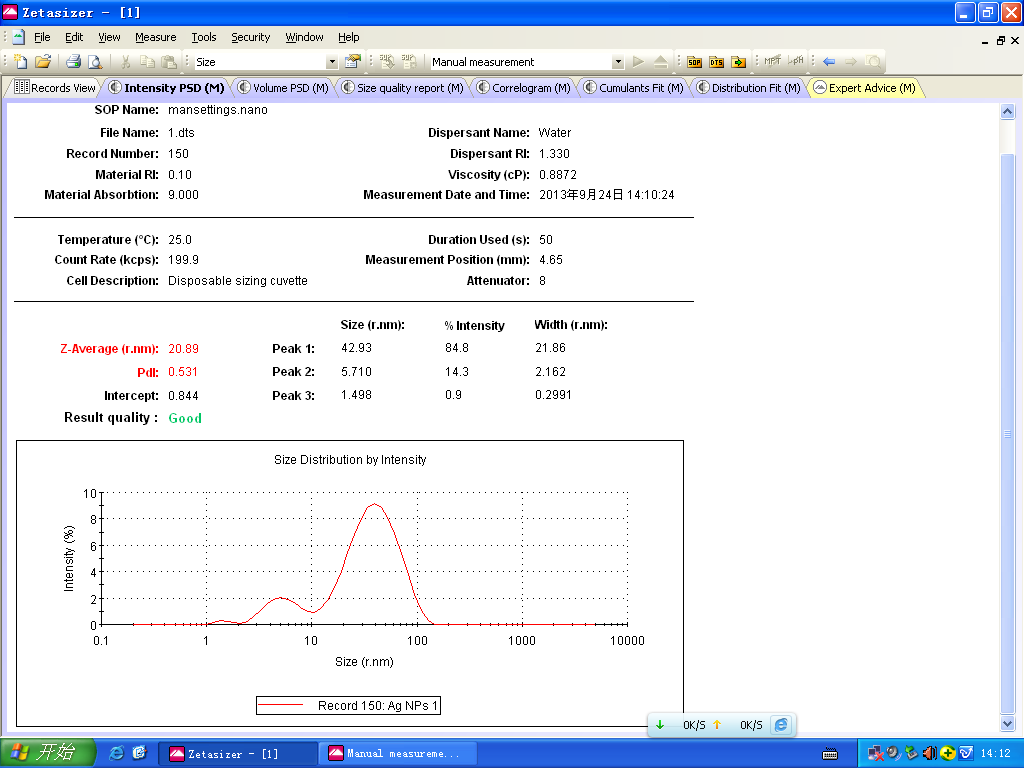


**Figure S2.** The size distribution of obtained Ag NPs colloid.

**Figure S3.** EDS pattern of S-g-C_3_N_4_/Ag.

**Figure S4.** UV-vis absorption spectrum of CV solution.

**Figure S5.** UV-vis absorption spectrum of the S-g-C_3_N_4_/Ag suspension.


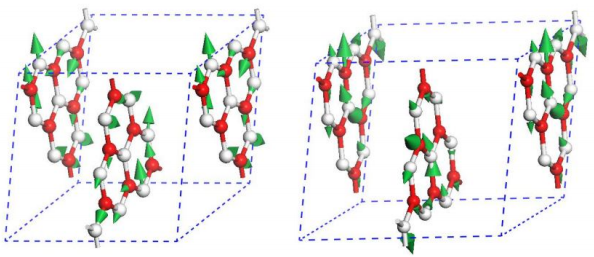


**Figure S6.** Assignments of two vibrational modes at 543 and 479 cm^-1^ of g-C_3_N_4_ based on CASTEP calculations. Red and gray spheres represent C and N atoms, respectively. The green arrows show dipole derivative unit vectors.
